# Rumen and Cecum Microbiomes in Reindeer (*Rangifer tarandus tarandus*) Are Changed in Response to a Lichen Diet and May Affect Enteric Methane Emissions

**DOI:** 10.1371/journal.pone.0155213

**Published:** 2016-05-09

**Authors:** Alejandro Salgado-Flores, Live H. Hagen, Suzanne L. Ishaq, Mirzaman Zamanzadeh, André-Denis G. Wright, Phillip B. Pope, Monica A. Sundset

**Affiliations:** 1 Department of Arctic and Marine Biology, UiT - The Arctic University of Norway, Tromsø, Norway; 2 Department of Chemistry, Biotechnology and Food Science, Norwegian University of Life Sciences, Ås, Norway; 3 Department of Animal and Range Sciences, Montana State University, Bozeman, Montana, United States of America; 4 University of Waterloo, Waterloo, Ontario, Canada; 5 School of Animal and Comparative Biomedical Sciences, University of Arizona, Tucson, Arizona, United States of America; Agriculture and Agri-Food Canada, CANADA

## Abstract

Reindeer (*Rangifer tarandus tarandus*) are large Holarctic herbivores whose heterogeneous diet has led to the development of a unique gastrointestinal microbiota, essential for the digestion of arctic flora, which may include a large proportion of lichens during winter. Lichens are rich in plant secondary metabolites, which may affect members of the gut microbial consortium, such as the methane-producing methanogenic archaea. Little is known about the effect of lichen consumption on the rumen and cecum microbiotas and how this may affect methanogenesis in reindeer. Here, we examined the effects of dietary lichens on the reindeer gut microbiota, especially methanogens. Samples from the rumen and cecum were collected from two groups of reindeer, fed either lichens (Ld: n = 4), or a standard pelleted feed (Pd: n = 3). Microbial densities (methanogens, bacteria and protozoa) were quantified using quantitative real-time PCR and methanogen and bacterial diversities were determined by 454 pyrosequencing of the 16S rRNA genes.

In general, the density of methanogens were not significantly affected (p>0.05) by the intake of lichens. *Methanobrevibacter* constituted the main archaeal genus (>95% of reads), with *Mbr*. *thaueri* CW as the dominant species in both groups of reindeer. Bacteria belonging to the uncharacterized *Ruminococcaceae* and the genus *Prevotella* were the dominant phylotypes in the rumen and cecum, in both diets (ranging between 16–38% total sequences). Bacteria belonging to the genus *Ruminococcus* (3.5% to 0.6%; p = 0.001) and uncharacterized phylotypes within the order *Bacteroidales* (8.4% to 1.3%; p = 0.027), were significantly decreased in the rumen of lichen-fed reindeer, but not in the cecum (p = 0.2 and p = 0.087, respectively). UniFrac-based analyses showed archaeal and bacterial libraries were significantly different between diets, in both the cecum and the rumen (vegan::Adonis: pseudo-F<0.05). Based upon previous literature, we suggest that the altered methanogen and bacterial profiles may account for expected lower methane emissions from lichen-fed reindeer.

## Introduction

Reindeer are large ruminants, widespread across the Northern Hemisphere. There are approximately five million animals that are taxonomically divided into seven extant subspecies [1]. Our focus is on the Norwegian reindeer (*Rangifer tarandus tarandus*), which accounts for ~ 200,000 animals, which are mainly herded in a nomadic system by the Saami people. Reindeer husbandry constitutes an essential part of the economy for the Saami community. Thus, gaining more insights on the nutritional physiology of these ruminants would be of high interest. Reindeer are classified as intermediate ruminant feeders [2,3]. In winter, when food is scarce and plants with poor nutritional value are available, reindeer may include a large proportion of lichens as a valuable extra energy source due to their high content of easily degradable carbohydrates [4–8]. In addition, lichens also possess a highly heterogeneous chemical composition with high contents of antimicrobial plant secondary metabolites (PSM) [7,9]. Severe toxic, or even lethal, effects have been reported after the consumption of lichens by sheep and elk (*Cervus canadensis*) [10,11]. However, reindeer can tolerate large proportions of this foodstuff in their diet.

Like other ruminants, reindeer rely on a highly complex, specialized microbiota, shaped by co-evolution with their Arctic diet, such as lichens, to allow the symbiotic microbial fermentation of plants in their rumen. This microbial consortium includes anaerobic bacteria, ciliated protozoa, fungi, and methanogenic archaea. Several studies have addressed the goal of characterizing the rumen microbial consortia [8,12–14]. However, little data are available on the microbiome of the cecum. Most of the degradation and fermentation of carbohydrates takes place in the rumen, but some material can still remain undigested before reaching the cecum, where further microbial digestion occurs [15,16]. Like the rumen, cecal digestion is also performed by specialized consortia of microorganisms, which produce volatile fatty acids (VFAs) that are absorbed by the host. This additional site of fermentation provides an extra carbon and energy source, which may influence the animal’s metabolism [17]. Accordingly, diet may also influence the microbiota housed in the large intestine. The characterization of this microbiota together with the rumen would allow a better understanding on the effects produced by the feeding regime on some metabolic processes, such as methanogenesis.

Enteric methane emissions from ruminants may result in not only an energy loss for the individual animal, accounting 2–12% of the total gross energy intake (GEI) [18], but is also a source of atmospheric contamination [19]. Among the different microbial groups, methanogens are the only methane-producing microorganisms in the rumen and cecum. They produce methane mainly by the reduction of carbon dioxide (and also acetate) with electrons taken mostly from hydrogen, with also formate and methyl compounds as minority electron sources [20]. The action of this microbial group is important for the maintenance of optimal anaerobic digestion [21,22]. In particular, this is mostly achieved via intimate hydrogen exchange with the other microbial groups (mostly the rumen ciliated protozoa) in the gut as high partial pressures of hydrogen may disrupt the anaerobic fermentation of polysaccharides degradation end products [23,24]. Several *in vitro* studies demonstrated a methane-suppressing effect exerted by some PSM (e.g. condensed tannins), via either direct inhibition on methanogens, or to any of their syntrophic partners [25,26]. The intake of forage high in PSM, or the addition of some of these compounds, led to depressed methanogenesis, as well as changes to the archaeal community profiles in sheep, goat and deer [27–29]. Nevertheless, very few studies have focused on relating the intake of diets high in PSM, or how this may alter the diversity of methanogens and their relationship with predicted lower methane emissions.

In the current study, we characterized the rumen and cecum microbiota from two groups of captive Norwegian reindeer fed two different diets (lichens, high in PSM, or standard pelleted feed). Based upon the extensive literature indicating a methane-suppressing effect with diets high in PSM, and considering the outstanding tolerance of reindeer to the intake of lichens, our objectives were as follows: (1) obtain a detailed approximation on how the rumen and cecum microbiota were affected by the ingestion of lichens, comparable to other PSM-rich diets; and, (2) to investigate how the alteration of these microbial profiles, especially methanogens in both the rumen and cecum, with the intake of lichens may potentially account for predicted low methanogenesis. In summary, significant differences were observed in the bacterial and archaeal microbiota at rumen and cecum level between both feeding regimes, with some specific archaeal phylotypes being altered with a lichen-based diet.

## Material and Methods

### Ethics statement

Most reindeer in Norway are owned and herded in a nomadic system by the Saami people, and animals used in this study originated from a privately owned herd gathered about 30 minutes from the city of Tromsø, where the University of Tromsø –The Arctic University of Norway (UiT)–is located. Animals were bought directly from the owner, and transported to our research facilities at UiT. Reindeer (*R*. *t*. *tarandus*) are not an endangered or a protected species, and no specific permission was required to buy and transport the animals to UiT. This project does not include field studies. All animal experiments were conducted after arrival to UiT, in approved animal research facilities at Department of Arctic and Marine Biology (permit no. 089). In general, the animals were maintained in large outdoor pens and fed ad libitum a pelleted feed, a lichen-based diet, or a mix of both depending on the season. The animals were sacrificed in a laboratory facility appropriate for that purpose, following a method of euthanasia approved by the National Animal Research Authority (no. 5399) by stunning with a bolt pistol and subsequent bleeding.

### Sampling

Whole rumen and cecum samples (n = 14; approx. 50 mL per sample) were collected from seven reindeer (NRruS = **N**orwegian **R**eindeer **ru**men **S**ample and NRceS = **N**orwegian **R**eindeer **ce**cum **S**ample) immediately after slaughter, and kept at -80°C. Samples were divided into two groups based on diet composition: samples 1–3 were collected from reindeer feeding on grass-based pellet concentrate (Pd) (23.3% oats, 18.9% timothy grass, 16% wheat bran and 11.2% barley). Samples 4–7 were collected from reindeer fed on a mix of different species of lichens (Ld) (mainly *Cladonia sterallis* sp) collected in southern Norway. The animals were fed with the corresponding diet for four weeks before slaughter and sample collection. Detailed conditions for each animal, such as gender, date of birth, body mass, weight after slaughter, as well as, rumen and cecum pH values at sampling are listed in Table 1.

**Table 1:** Anatomical and physiological conditions in the rumen and cecum of Norwegian reindeer at sampling.

Sample ID	Age	Sex	Body mass (kg)	Slaughter weight (kg)	Diet	Rumen pH	Cecum* pH
NRS1	Born Spring 2012	Female	59.6	34	Pellets/Conc.	6.63	6.76
NRS2	Born Spring 2012	Female	52.0	29	Pellets/Conc.	6.54	6.85
NRS3	Born Spring 2012	Female	54.4	29	Pellets/Conc.	5.94	6.53
NRS4	Born Spring 2007	Female	71.4	37	Lichen mix	5.58	5.74
NRS5	Born Spring 2003	Female	77.2	36	Lichen mix	5.57	6.04
NRS6	Born Spring 2010	Female	61.8	30	Lichen mix	6.04	6.09
NRS7	Born Spring 2003	Female	64.2	28	Lichen mix	5.53	5.63

*Significant differences in the pH values between both feeding regimes based on a permutation Welch’s t-test statistical analysis (P<0.05). Conc. = Concentrate.

### DNA extraction

Approximately 0.25 g aliquots from each partially thawed sample were used for the analyses. DNA extraction was performed following the Repeated Bead Beating plus Column (RBB+C) Method [30] with the following modifications. Stainless steel beads (Qiagen, Hilden, Germany) were used in place of sterile zirconia beads, and the incubation with DNase-free RNase was skipped (step 11). DNA was quantified using a NanoDrop 2000c spectrophotometer (Thermo Scientific, Waltham, MA, USA) and solutions were kept at -20°C until PCR amplification.

### Quantitative real-time PCR

Cell densities for each microbial group present in the DNA extracts from the different samples were estimated using quantitative real-time PCR. External standards were used for each microbial group. External standards for methanogens consisted in a purchased mix of *Methanobrevibacter smithii*, *Mbr*. *gottschalkii*, *Mbr*. *ruminantium* and *Mbr*. *millerae* (LGC Standards, Teddington, UK). Bacterial external standards was performed as described by Denman and McSweeney [31], using serial log dilutions of *Ruminococcus flavefaciens* ranging from 9.53 x 10^5^ to 9.53 x 10^8^ cells per mL. The external standards for protozoa ciliates were also prepared as reported by Skillman *et al*. [32], where protozoa cells were counted microscopically and serial dilutions were carried out to concentrations ranging from 1.86 x 10^3^ to 1.86 x 10^6^. DNA extractions from the serial dilutions for each microbial group were subsequently used as standards for the reactions. The primers used for these analyses are listed in Table 2. Experiments were performed in a BioRad CFX Thermocycler system (BioRad, Hercules, CA, USA) at the Department of Animal Science at the University of Vermont (USA). A total reaction volume of 25 μL, consisted of 12.5 μL of SYBR Green mix (Quanti- Tect^™^ SYBR^®^ Green PCR, Qiagen, Germany), 2.5 μL of each primer (400 μM) and 1 μL of DNA template (10 ng/ μL), and 6.5 μL of distilled water. PCR conditions were modified depending upon the microbial group tested (S1 File). A final melting curve analysis was performed after each experiment by continuously monitoring fluorescence signals from small increases of 0.5°C every 10s, in a temperature range from 60°C to 95°C, in order to check for primer specificity and discard DNA contamination. Calculations of threshold cycles (Ct) were automatically performed by the BioRad CFX manager software (v3.0). The logarithmic fraction of the resulting sigmoid-shaped curve after each reaction was used for calculations of the PCR efficiency, following the methods described by Liu and Saint [33]. Each DNA template was run in triplicate and C_t_ for only those reactions showing the highest efficiency (linear standard curve (R^2^) above 0.996) were included.

**Table 2:** List of the primers used in this study.

Technique	Target microbe	Primer pair	Sequence (5’ to 3’ direction)	References
Quantitative real-time PCR	Methanogens	qmcra-F	TTCGGTGGATCDCARAGRG C	[34]
		qmcra-R	GBARGTCGWAWCCGTAGAATCC	
	Bacteria	1114F	CGGCAACGAGCGCAACCC	[35]
		1275R	CCATTGTAGCACGTGTGTAGCC	
Diversity	Protozoa	PSSU-316F	GCTTTCGWTGGTAGTGTATT	[36]
		PSSU-539R	CTTGCCCTCYAATCGTWCT	
	Methanogens	340F	CCCTAYGGGGYGCASCAG	[37]
		1000R	GAGARGWRGTGCATGGCC	
	Bacteria	27F	AGAGTTTGATCCTGG	[38]
		519R	TTACCGCGGCTGCT	

### 16S rRNA amplicon library preparation

Bacterial and Archaeal PCR amplifications were performed in an Eppendorf Mastercycler Gradient (Eppendorf AG, Hamburg, Germany) in a total reaction volume of 25 μL. Reaction mixes consisted of 12.5 μL of iProof High-Fidelity Master Mix kit (BioRad, Hercules, CA, USA), containing 0.04 U/ μL of iProof DNA polymerase as well as 400 μM DNTPs. To each sample, 1.25 μL of Dimethyl sulfoxide (DMSO) was added in order to increase PCR efficiency, as well as 1 μL of each primer (400 nM) and 1 μL DNA template. 16S rRNA gene amplification was carried out using the bacterial primer set 27F and 515R, targeting the variable regions V1-V3 and yielding a 500-base pairs (bp) size amplicon product; and the archaeal primer set 340F and 1000R, producing a 660-bp size product (Table 2). Each primer contained one of the Life Sciences adaptors (adaptor A on the reverse primer and adaptor B on the forward primer). In addition, an 8-nucleotide (nts) multiplex identifier (MID) [39] was present downstream on the reverse primer to identify sequence reads for bioinformatics analyses.

Conditions for PCR reactions were as follows: an initial denaturation step at 98°C for 30 s; 25 or 35 cycles for bacterial or archaeal primer sets, respectively, consisting of denaturation at 98°C for 10 s; annealing at 60°C or 58°C for bacteria or archaea, respectively, for 30 s; and extension at 72°C for 45 s. A final extension step at 72°C for 7 min was run and samples were kept at 4°C until checked by 1% agarose gel electrophoresis. DNA concentration for each sample was quantified using a Qubit fluorometer (Invitrogen, Carlsbad, CA, USA), samples pooled in equimolar amounts and checked by 1% agarose gel electrophoresis. Resulting bands from the pooled samples were finally excised from gel and purified using the NucleoSpin Gel and PCR Clean-up kit (Macherey-Nagel, Düren, Germany), following manufacturer’s protocol. Purified amplicons were then stored at -20°C until sequencing. Sequencing was performed by 454/Roche GS FLX, LIB-L chemistry, at the Norwegian Sequencing Centre (NSC), in Oslo.

### Sequence processing

Resulting bacterial and archaeal 16S rRNA sequences were analyzed using the Quantitative Insights Into Microbial Ecology (QIIME) pipeline [40]. A first filtering step was performed to assure quality. Sequences were discarded for the following reasons: total length fell out of 350–650 nts; homopolymer runs exceeded 6 bases; average quality score resulted less than 25; and a mismatch in the primer sequences occurred. Sequences were clustered as Operational Taxonomic Unit (OTU) based on a 97% similarity criterion with the QIIME-incorporated version of USEARCH [41] with a word length of 64. Any sequence flagged as putative chimera was identified with UCHIME [42], and finally discarded from the analysis.

### Sequence analysis

OTU-representative sequences were chosen based on sequence abundance and subsequently aligned against a Greengenes core-set reference database [43], with the Python-based version of the Near Alignment Space Termination (NAST) algorithm [44] in QIIME. Taxonomic identification down to genus level for all the previously aligned sequences was performed using the RDP classifier [45], at a default 80% confidence cut-off, and where a Naïve-Bayesian algorithm is applied against the RDP-II reference database. Classification at species level for the Archaeal sequences was performed using the Basic Local Alignment Search Tool (BLAST) software [46] from the National Center for Biotechnology Information (NCBI) website. Alpha-diversity estimators assessing OTU richness (CHAO) [47], sample evenness (Shannon) [48], sample coverage (good’s coverage) [49] and total observed OTUs (i.e, observed_species) were calculated after random subsampling of the different datasets. Resulting rarefaction curves were generated with the make_rarefaction_plots.py script. In addition, pairwise sample dissimilarity analyses (beta-diversity) were performed using subsampled datasets adjusted to the one yielding the lowest counts in order to avoid any potential bias. Principal coordinate analysis (PCoA) plots were created using pre-calculated weighted UniFrac distance matrices. Analyses were performed separately based on microbial target (bacteria and archaea) and sampling site (rumen and cecum).

### Functional prediction on metagenomes

Bacterial and archaeal gene contents of described metagenomes for each sample were predicted using PICRUSt [50] online version available in the online Galaxy platform (https://huttenhower.sph.harvard.edu/galaxy/). Firstly, a closed reference OTU table was created using sequence datasets obtained after quality check with QIIME (as previously described), with a Greengenes core set reference database. The resulting closed reference OTU table was then normalized based on 16S rRNA gene copy number prior to metagenome prediction, and subsequently categorized by function based on the Kyoto Encyclopedia of Genes and Genomes (KEGG) pathways in Galaxy online. Statistical analysis and plot generation of the resulting biom file was performed with STAMP v2.0.9 [51]. In particular, pairwise comparison of the KEGG pathways between both types of diets was performed applying a Welcht’s (two-tailed) *t*-test with 95% confidence intervals. KEGG pathways displaying a *p*-value below 0.05 were considered as statistical significant.

### Volatile fatty-acids chemical analysis

The concentrations of VFAs in reindeer rumen samples were analyzed using a high performance liquid chromatography (HPLC) (Dionex Ultimate 3000) equipped with a C-18 column (Agilent eclipse plus C-18; 3.5 μ; 2.1×150mm) and a UV detector set at 210 nm. The temperature of the column compartment was set at 40°C. The samples were loaded on a Dionex autosampler (Ultimate 3000). Before analysis, the samples were centrifuged at 14,000 rpm for 10 min and were then filtered through a 0.45 μm syringe filter. The pH of the samples was measured to assure the pH was less than 2.5. If not, then the pH was adjusted using sulfuric acid. Methanol (100%) and 2.5 mM sulfuric acid were used as eluents at a flow rate of 0.3 mL/min. VFAs including acetic, propionic, iso-butyric, n-butyric were measured during a 30-min run. Iso-butyric acid was not detected in any of the samples and thus it was finally discarded from the analysis.

### Statistical analysis

Two-tailed Student’s *t*-test was used to check for significant differences between the results obtained with quantitative real-time PCR from the different feeding regimes. In addition, permutation Welch’s *t*-test [52] (9,999 permutations) analyses were performed to evaluate significance between similar phylotypes from different feeding regimes, using the ‘coin’ and ‘perm’ packages in ‘R’ (https://cran.r-project.org/mirrors.html). Significance for PCoA (beta-diversity) analyses was tested with multivariate permutation tests using the nonparametric method ‘Adonis’ (999 permutations) included in the package ‘vegan’ of the QIIME-incorporated version of ‘R’. Tests were performed after random subsampling of datasets from each sample taking a fixed number (depth) of sequences (2,000 sequences /sample). All the analyses were performed separately for bacteria and archaea as well as sampling site (rumen or cecum).

## Results

### Quantitative real-time PCR results in rumen and cecum samples

Population densities (cells per gram wet weight (cells/gww)) of the different microbial types (methanogens, bacteria and protozoa) found in rumen and cecum samples from both groups of reindeer were determined using quantitative real-time PCR (Tables 3 and 4). In summary, no significant differences were obtained comparing the number of any of the three microbial groups, regardless of feeding regime, or sampling site. Methanogens densities varied from a mean 1.7 x 10^7^ to 8.86 x 10^6^ cells/gww in rumen samples from lichen-fed reindeer (p = 0.481). In cecum samples these numbers constituted an average 2.56 x 10^5^ and 2.32 x 10^6^ cells per gram of wet weight with pellets and lichens, respectively (p = 0.06). Two cecal samples from the reindeer fed with pelleted feed (NRceS2 and NRceS3) yielded no signal for methanogens in the tests (Table 4). Similarly, one cecal sample from one of the reindeer, fed with pelleted feed (NRceS2), yielded no signal for protozoa ciliates. Rumen protozoa accounted for an average 3x10^7^ and 4.92x10^6^ cells per gram of wet weight for pellet and lichen fed reindeer, respectively. In cecum samples these numbers were 1.08x10^3^ (pelleted diet) and 1.08x10^4^ (lichen diet) cells/gww. As indicated, no significant differences were observed between the densities of protozoa from samples of reindeer fed with pellets, or lichens, in any of the sampling sites (rumen: p = 0.265; cecum: p = 0.095). Finally, bacterial counts remained practically unaltered between the two diets, and independently of the two sampling sites (Tables 3 and 4) (rumen: p = 0.731; cecum: p = 0.436). This microbial group showed the highest values in the rumen and cecum compared to the other two microbial groups (methanogens and protozoa), with average densities as high as x10^8^cells/gww.

**Table 3:** Concentration of methanogens, bacteria and protozoa in the rumen of Norwegian reindeer.

Animal	Methanogens	Bacteria	Protozoa	Diet
NRruS1	1.13x107	9.84x10^8^	5.6x10^7^	Pellets
NRruS2	8.51x10^6^	8.08x10^8^	4.02x10^6^	Pellets
NRruS3	3.11x10^7^	7.19x10^8^	3x10^7^	Pellets
Mean(SE)	1.7x10^7^ (1.23x10^7^)	8.37x10^8^ (1.35x10^8^)	3x10^7^ (2.6x10^7^)	Pellets
NRruS4	5.88x10^6^	4.30x10^8^	1.04x10^4^	Lichens
NRruS5	6.3x10^5^	1.7x10^9^	2.78x10^7^	Lichens
NRruS6	2.97x10^6^	1.46x10^8^	2.39x10^4^	Lichens
NRruS7	2.89x10^6^	5.41x10^8^	8.83x10^5^	Lichens
Mean(SE)	8.86x10^6^ (1.02 x10^7^)	3.84x10^8^ (1.33x10^8^)	4.92x10^6^ (1.12 x10^7^)	Lichens

Microbial populations were determined by qrtPCR. Total counts are given based on whole rumen contents (number of cells per gram wet weight).

SE = Standard error

**Table 4:** Concentration of methanogens, bacteria and protozoa in the cecum of Norwegian reindeer.

Animal	Methanogens	Bacteria	Protozoa	Diet
NRceS1	ND	5.44x10^6^	ND	Pellets
NRceS2	ND	6.45x10^8^	2.04x10^3^	Pellets
NRceS3	7.69x10^5^	4.14x10^8^	1.2x10^3^	Pellets
Mean (SE)	2.56x10^5^ (4.44x10^5^)	3.55x10^8^ (3.24x10^8^)	1.08x10^3^ (1.02x10^3^)	Pellets
NRceS4	2.09x10^6^	3.45x10^8^	2.39x10^3^	Lichen
NRceS5	4.92x10^6^	5.82x10^8^	3.12x10^3^	Lichen
NRceS6	1.59x10^6^	2.99x10^8^	2.56x10^4^	Lichen
NRceS7	6.83 x10^6^	3.11x10^8^	1.21x10^4^	Lichen
Mean (SE)	2.32x10^6^ (1.83 x10^6^)	3.84x10^8^ (1.33x10^8^)	1.08x10^4^ (1.08x10^4^)	Lichen

Microbial populations were determined by qrtPCR. Total counts are given based on whole cecum contents (number of cells per gram wet weight).

SE = Standard error

ND = not detected. C_t_ values lower than 35 cycles.

### Taxonomic identification

#### Bacterial 16S rRNA gene sequence analyses in rumen samples

In total, 117,774 bacterial 16S rRNA sequences were retrieved from the rumen samples collected in both groups of reindeer (fed pellets and lichens), with numbers varying from 14,528 to 19,055 sequences per sample. Quality check and trimming down to 500 bases in length resulted in 97,633 high quality sequences used for downstream analyses. OTU-clustering based on a 97% similarity criterion yielded 2,290 chimera-free OTUs, with 341 OTUs being shared between both feeding regimes. Identification at phylum level with RDP classifier tool showed *Firmicutes* and *Bacteroidetes* as the two most representative phyla in both groups (S2 and S3 Files). As much as 49.6% and 40.5% of total 35,012 sequences (on average) were assigned to *Firmicutes* and *Bacteroidetes*, respectively, in reindeer fed with pellets concentrate. *Firmicutes*-associated phylotypes constituted an average of 63.7% of 62,621 of total sequences in reindeer fed with lichens, whereas in reindeer fed pellets this group resulted in a 49.6%. In contrast, *Bacteroidetes* phylotypes accounted for an average of 40.5% of total sequences in reindeer fed pellets and 29.7% of total sequences in reindeer fed a lichen-based diet. Despite the differences observed in the relative abundance of these phyla between both groups of reindeer, statistically the difference was not significant for any of the samples (Permutation Welch’s *t*-test: *Firmicutes*: p = 0.196; *Bacteroidetes*: p = 0.344).

**Fig 1:**
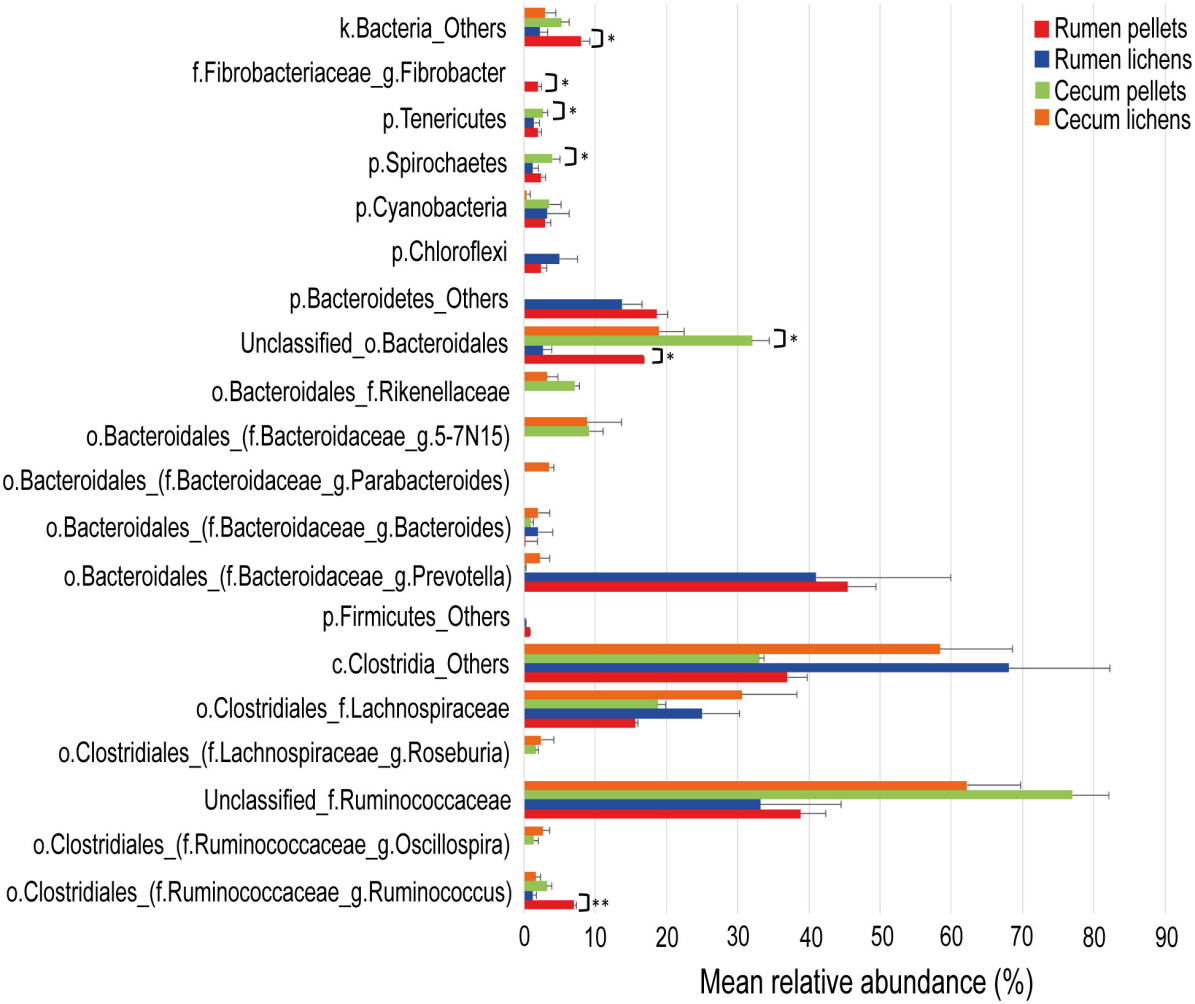
Fluctuations on the rumen and cecum bacterial microbiota in lichen-fed Norwegian reindeer. Mean values for the total 16S rRNA gene sequences assigned to each phylotype are displayed, with taxonomical classification down to genus level. Standard error (black lines) and statistical significance (asterisk symbol; p<0.05) obtained with permutation Welch’s *t*-test (9999 permutations) analysis are also included. Only samples from the same sampling site were used for statistical comparisons (i.e. rumen or cecum). Rumen sample from reindeer fed pellets (Rumen pellets) (red); Rumen-lichens (blue); Cecum pellets (green); Cecum lichens (orange).

Classification down to genus level also agreed with the differences described above (Fig 1, S2 and S3 Files). Within the *Firmicutes*, the relative proportion of uncharacterized genera belonging to the class *Clostridia* represented an average 18.5% and 34.0% of total sequences with the intake of pellets or lichens, respectively (p = 0.198). Similarly, unclassified genera within the family *Lachnospiraceae* accounted for an average 7.8% of total sequences in reindeer fed with pellets and 12.5% with lichens (p = 0.2). *Ruminococcus* spp. (Pd: 3.5%; Ld: 0.6%) were significantly reduced (p = 0.001) by the intake of lichens and accounted for a 2.9% of the reads. Within the *Bacteroidetes*, only unclassified genera of the order *Bacteroidales* were significantly influenced (p = 0.027) by diet composition, decreasing from an average 8.4% to 1.3% of total sequences with lichen as the only sustenance. Members of the cellulose-degrading *Fibrobacteres* phylum were only present in reindeer fed pellets, which accounted 1% of the total sequences, on average.

#### Bacterial 16S rRNA sequence analyses in cecum samples

Cecum samples yielded 60,693 raw bacterial 16S rRNA gene sequences resulting in a final 46,845 high quality sequences after trimming to 500 bases. A total of 1,291 chimera-free OTUs were subsequently obtained with 210 OTUs shared between both groups. Similarly, as in rumen samples, *Firmicutes* and *Bacteroidetes* were the two major phyla, regardless of diet composition (S2 and S4 Files). The average percentage of total *Firmicutes*-related phylotypes were 67.3% and 79.2% of a total 12,648 and 34,197 sequences in pellet-fed and lichen-fed reindeer, respectively. *Bacteroidetes* represented an average 24.6% in reindeer fed pellets and 19.5% with lichens as the only sustenance. In both cases, the differences observed between both groups of reindeer, for these two phyla, were not significant (*Firmicutes*: p = 0.117; *Bacteroidetes*: p = 0.310).

Classification down to genus level showed uncharacterized phylotypes related to the family *Ruminococcaceae* as the major bacterial group in both groups of reindeer (Pd: 38.5%; Ld: 31.1% of total sequences on average), followed by phylotypes assigned to the order *Clostridiales* (Pd: 16.5%; Ld: 29.2% total sequences). Unclassified genera belonging to the family *Lachnospiraceae* resulted in an average 9.4% and 15.3% of total sequences in reindeer fed with pellets and lichens, respectively (p = 0.261). A similar trend was obtained for unclassified *Clostridia*-related phylotypes, which accounted for an average 16.5% of total sequences in pellets-fed reindeer and 29.2% in those fed with lichens (p = 0.09) (Fig 1, S2 and S4 Files). Instead, uncharacterized phylotypes belonging to the order *Bacteroidetes* experienced a decrease (p = 0.087) in lichen-fed reindeer (Pd: 16%; Ld: 9.5%). Phylotypes assigned to the phyla *Spirochaetes* (Pd: 2%; Ld: 0%) and *Tenericutes* (Pd: 1.3%; Ld: 0%) were significantly decreased in reindeer offered a lichen-based diet (Fig 1, S2 and S4 Files).

#### Archaeal 16S rRNA sequence analyses in rumen samples

**Fig 2:**
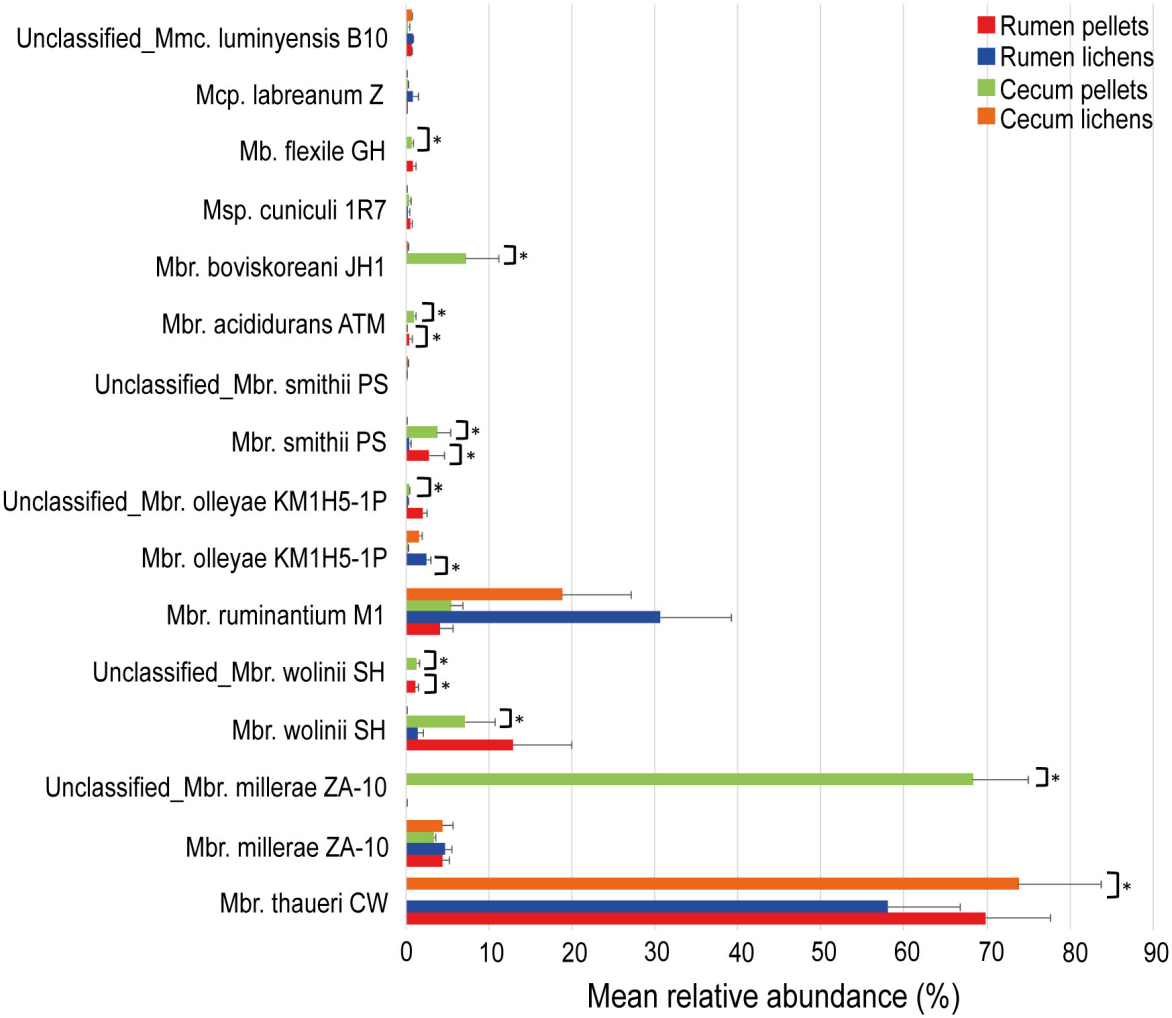
Fluctuations on the rumen and cecum archaeal microbiota in lichen-fed Norwegian reindeer. Mean values for the total 16S rRNA sequences assigned to each phylotype are displayed, with taxonomical classification at species/strain level. Standard error (black lines) and statistical significance (asterisk symbol; p<0.05) with permutation Welch’s *t*-test (9999 permutations) analysis were also included. Only samples from the same sampling site were used for statistical comparisons (i.e. rumen or cecum). Rumen sample from reindeer fed pellets (Rumen pellets) (red); Rumen-lichen (blue); Cecum pellets (green); Cecum lichen (orange).

Overall, 78,201 Archaeal 16S rRNA sequences were retrieved from the seven rumen samples resulting in 75,739 after quality check and trimming down to 500 bases. Numbers of sequence per sample ranged 7,682 to 15,780 and yielded a total of 53 chimera-free OTUs, with as many as 49 OTUs shared by both groups of reindeer. In general, *Euryarchaeota* was the only phylum found in all the samples (S5 and S6 Files). At the genus level, *Methanobrevibacter* spp. accounted for the majority of the sequences, independent of the feeding regime (Pd: 97.8% of 32,144 total sequences; Ld: 98.1% of 43,595 total sequences). Taxonomical identification down to species/strain level (≥97% similarity with GenBank database representatives) showed phylotypes sharing a 98% similarity to *Methanobrevibacter thaueri* strain CW constituting the major archaeal taxa found in both groups of reindeer (Pd: 69.1%; Ld: 58.2% of total sequences, on average) (p = 0.35) (Fig 2, S5 and S6 Files). Phylotypes identified as *Methanobrevibacter wolinii* strain SH-related OTUs (98% similarity) represented the second most prevalent phylotype in the pellets-based group (13.4% total sequences), whereas they accounted for only an average 1.5% in reindeer fed with lichens. However, this variation was not significant (p = 0.092). A significant decrease was observed in members assigned to *Methanobrevibacter smithii* strain PS (97% similarity) in lichen-fed reindeer (Pd: 2.8%; Ld: 0.4%. p = 0.029). OTUs sharing a 99% similarity to *Methanobrevibacter ruminantium* strain M1 constituted the second most prevalent phylotype in reindeer fed with lichens (30.8% total sequences), but only an average 4.2% in pellets-fed reindeer. Despite the variation observed for this phylotype, between both groups of reindeer, no statistical significance was obtained (p = 0.054). Finally, phylotypes related to *Methanobrevibacter olleyae* strain KM1H5-1P (97% similarity) significantly increased in lichen-fed reindeer (Pd: 0.0%; Ld: 2.6%) (p = 0.030).

#### Archaeal 16S rRNA sequence analyses in cecum samples

A total of 73,977 Archaeal 16S rRNA sequences were generated from cecum samples, resulting in a final 71,498 sequences after quality filtering and trimming. Sequences were assigned to a total 47 OTUs with as much as 46 OTUs shared by all the samples. Similar to the rumen samples, phylotypes belonging to the phylum *Euryarchaeota* were the only microbes detected in all samples, with most of the OTUs belonging to the genus *Methanobrevibacter* (Pd: 96.7% of 32, 141 sequences; Ld: 99.2% of 39,357 sequences, on average) (Fig 2, S5 and S7 Files). At strain/species level, an average 68.1% of total sequences were identified as *Methanobrevibacter millerae* strain ZA-10 in reindeer fed with pellets, but sharing less than 97% identity with this methanogens. Instead, in lichen-fed reindeer, phylotypes sharing a 98% similarity to *Methanobrevibacter thaueri* strain CW constituted the major phylotype (73.8% total sequences, on average) (Fig 2, S5 and S7 Files). Other phylotypes, showing a significant decrease with a lichen-based diet, were *Methanobrevibacter wolinii* strain SH (98% similarity) (Pd: 7.1%; Ld: 0.1%; p = 0.027) and *Methanobrevibacter boviskoreani* strain JH1 (98% similarity) (Pd: 7.2%; Ld: 0.2%. p = 0.028). Phylotypes sharing a 99% similarity with *Methanobrevibacter ruminantium* strain M1 resulted in 5.4% and 18.8% of total sequences in reindeer fed with pellets and lichens, respectively (p = 0.258). (Fig 2, S5 and S7 Files). The same trend was observed for phylotypes identified as *Mbr*. *olleyae* strain KM1H5-1P (97% similarity) (Pd: 0.2%; Ld: 1.6% total sequences. p = 0.058).

### Whole community comparisons

**Fig 3:**
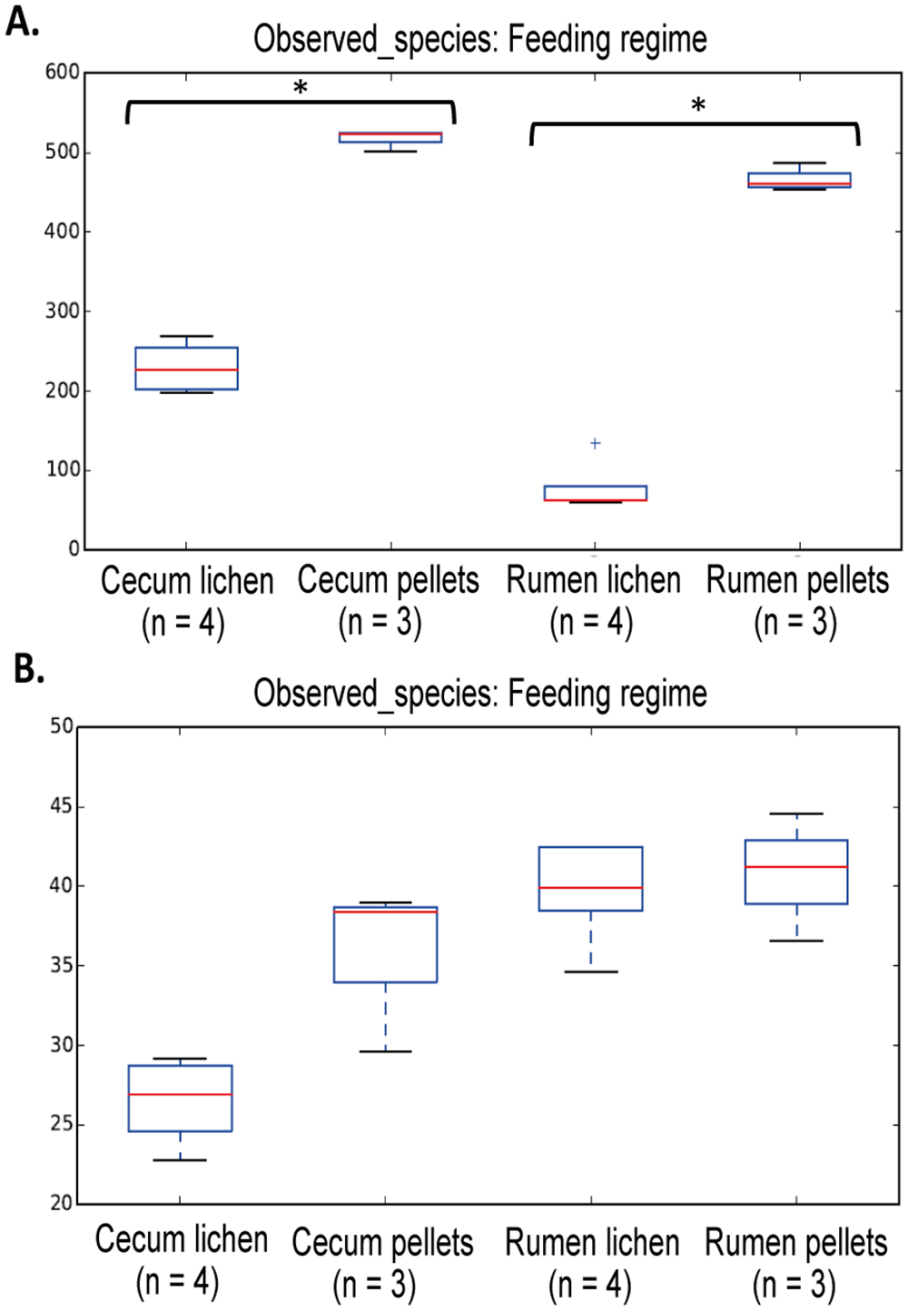
Box-plots within-sample community diversity comparisons from rumen and cecum samples in Norwegian reindeer. (A) Mean alpha diversity values for total unique OTUs (observed_species) calculated for the bacterial fraction of the microbiome. (B) Mean alpha diversity values for total unique OTUs (observed_species) calculated for the archaeal fraction of the microbiome. Box-plots were calculated using average values obtained from randomly subsampled datasets for each sample with a sample depth of 2000 sequences and 10 iterations at each subsampling step. Pairwise comparisons were performed only between samples from the same sample site (rumen or cecum) and statistical significance (asterisk symbol; p<0.05) was calculated with non-parametric t-test with Monte Carlo permutations (n = 999).

**Fig 4:**
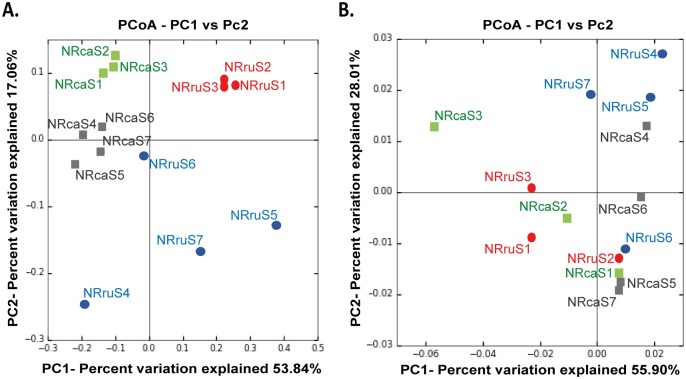
Principal coordinate analysis of the microbial community structure from rumen and cecum in Norwegian reindeer. (A) PCoA plot illustrating the bacterial community structures. (B) PCoA plot illustrating the archaeal community composition. PCoA plots were generate based on weighted UniFrac distance matrices. Colour style and labelling refers to the type of diet provided and sample origin: rumen samples from reindeer fed pellets (rumen-pellets) (red dots); rumen-lichens (blue dots); cecum-pellets (green squares); cecum-lichens (dark-grey squares). Statistical comparisons were performed only between samples from the same sampling site (rumen or cecum).

In general, the total number of unique bacterial OTUs, taken as an indicator of sample diversity, was significantly decreased (Monte Carlo distance-based t-test, p<0.05) when reindeer were fed with a lichen-based diet (Fig 3a, S1 Table). Between-sample comparisons of the different microbial libraries (beta diversity) illustrated by weighted UniFrac-based PCoA plots showed those samples belonging to the same feeding regime (pellets or lichens) grouping together in both sampling sites (rumen and cecum) (Fig 4a). Non-parametric Adonis tests (Rumen samples: pseudo-F = 0.037; Cecum samples: pseudo-F = 0.032) further corroborated these results, thus indicating that bacterial profiles from reindeer fed pellets or lichens were different, in both rumen and cecum.

No significant differences were observed on any alpha diversity parameter in the archaeal datasets (Fig 3b, S2 Table), except species richness (chao1), which was negatively influenced in cecum samples from reindeer fed lichens (Monte Carlo distance-based t-test, p = 0.04). PCoA plots showed a more scattered sample distribution than in bacteria (Fig 4b). However, UniFrac-based Adonis statistical tests (Rumen samples: pseudo-F = 0.029; Cecum samples: pseudo-F = 0.028) indicated significantly different archaeal profiles in rumen and cecum samples between both groups of reindeer.

### Predicted microbiome function

**Fig 5:**
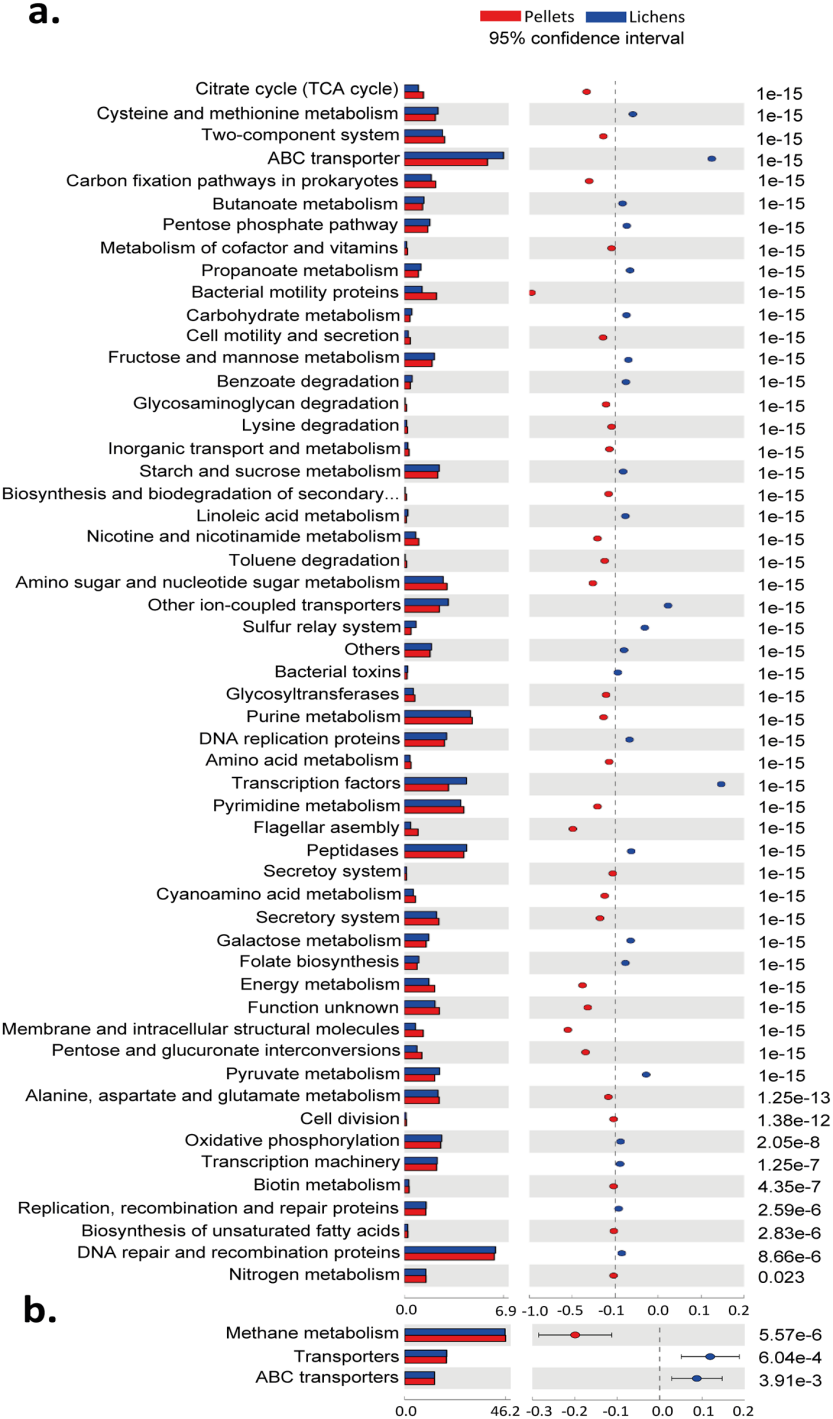
Comparisons of imputed metagenome prediction of the bacterial and archaeal metagenomes in Norwegian reindeer. Relative abundances for each KEGG metabolic pathway present in each metagenome were calculated and plotted with STAMP. KEGG pathways that were significantly different (IC: 95%. *p*-value <0.05) between diets for (A) bacterial and (B) archaeal predicted gene functions are showed. Color pattern is set based on diet composition: pellets concentrate (red); lichens (blue).

As observed, the different diets led to diverse microbial community structures in bacteria and archaea as well as in the two different digestive compartments. Accordingly, variations in the functional gene contents for each metagenome can also be assumed. In the absence of shotgun metagenomic sequencing data, we performed a first approximation to the gene content in our 16S rRNA libraries applying PICRUSt. Briefly, PICRUSt is used to predict present gene families from 16S rRNA gene data and a reference database applying evolutionary modelling. Relative abundances for imputed KEGG pathways were calculated so that any potential changes in the overall metabolic functions predicted to libraries from animals fed either pellets or lichens were assessed. Predicted genes related to several KEGG pathways, such as pyruvate and carbohydrate metabolism, as well as those pathways involved in fatty acid metabolism (propanoate and butanoate metabolism), showed a significantly higher relative abundance (p = 1.0e-15) with the consumption of lichens (Fig 5a). Gene predictions for starch and sucrose metabolism KEGG pathways involving metabolic routes for several polysaccharides like xylan, pectin, cellulose, and beta-glucan were also positively influenced on a lichen-based diet (p = 2.22e-15). In contrast, archaeal gene contents directly involved in methane metabolism were present in a significantly lower relative proportions in lichen fed reindeer (p = 1.0e-15) (Fig 5b).

### Volatile fatty acids chemical analysis

Total concentrations of some VFAs, such as acetate, propionate and n-Butyrate from rumen samples, in both groups of reindeer, were determined by HPLC. In summary, no statistical differences were observed in the average concentrations of acetate (Pd: 35.4 mg/mL; Ld: 30.8 mg/mL. p = 0.696) or n-Butyrate (Pd: 4 mg/mL; Ld: 4.9 mg/mL. p = 1) (S3 Table). Nonetheless, propionate displayed the highest fluctuation observed between diets with a considerable decrease from an average 48.2 mg/mL to 11.8 mg/mL in reindeer fed with lichens, although this difference was not statistically significant (p = 0.097).

## Discussion

### Fluctuations on microbial density by the ingestion of lichens

Rumen counts for the three microbial groups (bacteria, archaea and ciliate protozoa) tested in all samples were generally lower than those reported for free-ranging Norwegian reindeer [13]. This may likely be attributed to the nutritional constraints of a controlled diet in comparison to highly varied natural pastures, rich in proteins and minerals [5]. Both *in vitro* and *in vivo* studies indicated a high sensitivity to PSM by ciliates [25,53] and methanogens [26]. However, resistance has also been described in both microbial groups [54,55]. This resistance may potentially explain the lack of effect observed in the total numbers of methanogens and ciliates in the rumen and cecum of reindeer fed with lichens. Another potential explanation may be related to the PSM-degrading capacity reported for several rumen bacterial isolates from reindeer [56], which may attenuate the negative effects exerted by these compounds on the rumen and cecum microbiota in lichen-fed reindeer.

### Bacterial diversity permutations with a lichen-based diet

In summary, *Bacteroidetes* and *Firmicutes* were the major phylotypes found in reindeer, offered either diet, and from both sampling sites. Although in general, *Firmicutes* was present at a higher proportion in cecum samples. These two phyla commonly dominated the gastrointestinal tract of free-ranging and captive ruminants [14,57,58]. The proportion of these two phyla depends on the type of substrates they are exposed to. For example, *Bacteroidetes* phylotypes are mostly associated with the presence of easily fermentable carbohydrates (i.e. starch), and proteins. Whereas, some *Firmicutes*-related members are involved in the degradation of recalcitrant substrates, such as cellulose [59]. Considering their divergent metabolic strategies, the proportion of these two phyla (i.e. *Firmicutes* to *Bacteroidetes* (F-B) ratio) will be affected by the polysaccharides found in the diet, which may partly affect the host metabolism via VFAs production from fermentation. No significant differences were observed between the F-B ratios obtained from each diet, in either the rumen (p = 0.143), or the cecum (p = 0.107) (Fig 1; S2 File). Nonetheless, UniFrac-based community analyses showed overall bacterial profiles significantly differed between both feeding regimes independently of the sampling site (pseudo-F<0.05). Reindeer in the present study were supplied with a mixture of lichens of the genera *Cladonia* and *Cetraria*, which are composed of several polysaccharides (hemicellulose, xylan, lichenin) structurally dissimilar to those commonly found in vascular plants [7]. Any existing disparity in the structural nature of these polysaccharides between both diets may partially account for the differences observed in their respective bacterial community from both anaerobic chambers.

PSM, which are found at a high proportion and variety in lichens, has extensively been reported to possess a disrupting effect on several bacterial phylotypes [26,54]. For instance, *in vitro* assays indicated a dose-dependent altered endoglucanase and proteolytic activities in the presence of condensed tannins for several *Firmicutes* species, in addition to growth inhibition at high concentrations in *Ruminococcus albus* [60]. This inhibitory effect on *Ruminococcus* spp. may explain the significant reduction in the relative proportion of this family obtained in the rumen of reindeer fed with lichens (Fig 1; S2 and S3 Files). Aagnes *et al*. [8] described the rumen microbiome in reindeer fed, *ad libitum*, a lichen-based diet and showed an increase in bacteria belonging to the phylum *Firmicutes*, like *Clostridium* and *Streptococcus*. Furthermore, *in vitro* assays with *Clostridia*-related isolates from the rumen of Norwegian reindeer also indicated degradation of some plant anti-nutrients (e.g. tannins), together with enhanced growth [56,61]. Nonetheless, a negative, or absent, response to the presence of tannins was also reported in *in vitro* studies, with members belonging to the class *Clostridia* [62,63], which may account for the lack of differences obtained between both groups of reindeer (rumen: p = 0.198; cecum: p = 0.090).

*Prevotella* spp. was also reported as the dominant genus within the phylum *Bacteroidetes* in the rumen microbiome of Chinese sika deer fed with an oak leaves-based diet, also high in PSM [29]. This genus may be able to tolerate and thrive under diets enriched in the antimicrobial PSM through an unknown mechanism, which may explain for their comparable relative proportions found in both diets. In contrast, a growth-suppressing effect has been described for some *Bacteroides* spp. in *in vitro* studies with phenolics and aromatic compounds extracted from tea [64]. This suppressing effect may explain for the significant decrease observed in unclassified *Bacteroidales*-related phylotypes in lichen-fed reindeer, in both anaerobic chambers (Fig 1; S2, S3 and S4 Files). In addition, a general decrease in diversity was observed in the rumen and cecum of reindeer fed with lichens. This observation agrees with decreased diversity reported in rumen samples from Chinese sika deer fed with a diet high in PSM [29].

### Altered archaeal diversity and potential effects on methanogenesis

Similar to bacteria, the archaea were significantly different in reindeer from the two dietary groups (Fig 4b), with *Methanobrevibacter* spp. representing the major archaeal phylotypes in all the samples (Fig 2; S5 File). *Methanobrevibacter* was also found to be the dominant genus in free ranging reindeer (summer pastures) and cattle (high fiber diet) [13,65]. A two point five-fold increase in the abundance of members belonging to this genus has been described for cattle associated to high methane outputs compared to cattle yielding low-methane emissions [66]. In the present study, no significant differences were observed in the proportion of *Methanobrevibacter* spp. between reindeer fed with pellets, or lichens (S5 File; p = 0.9). Although these results may hint to unaltered methane emissions from reindeer, fed lichens, several *in vivo* and *in vitro* studies have described a strong negative effect on methanogenesis by PSM [25,26].

In some instances, the diversity of methanogens at strain level play key roles in affecting the methane production rather than the density of methanogens [67,68]. *Methanobrevibacter thaueri* is common in the rumen of several ruminants, such as reindeer and cattle with no diet specificity [13,69]. Up to now, only one study found that *Mbr*. *thaueri* was the dominant phylotype in the rumen of wild Impalas (*Aepyceros melampus melampus*) [70]. King *et al*. [71] suggested a classification method for methanogens based on phylogenetic distribution and representation, with *Mbr*. *smithii*, *Mbr*. *gottschalkii*, *Mbr*. *millerae* and *Mbr*. *thaueri* (SGMT-group) and *Mbr*. *ruminantium* and *Mbr*. *olleyae* (RO-group) divided in two separate clades. Although, *Mbr*. *gottschalkii* was not present in any samples, the SGMT:RO ratio was generally lower in reindeer fed with lichens, although these variations were not significant (Rumen: Pd = 12.1; Ld = 1.9. p = 0.129; Cecum: Pd = 12.4; Ld = 3.8. p = 0.9) (Fig 2, S5 File). Two of these methanogens, *Mbr*. *smithii* strain PS and *Mbr*. *olleyae* strain KM1H5-1P, were significantly altered with the intake of lichens, showing a decrease and an increase, respectively. A similar trend was obtained in cecum samples, although it was not statistically supported (p = 0.058). Terminal restriction fragment length polymorphism (t-RFLP) data, relating methanogenesis with methanogen community structure in Swedish dairy cows, suggested that methane emissions were positively correlated with dominance by the SGMT-clade over the RO-clade methanogens [72]. These results may suggest that increased relative proportion of RO-methanogens (or decreasing members of the SGMT clade) related to animals with lower methane yields. In conjunction with a significant increase in *Mbr*. *olleyae* strain KM1H5-1P, *Mbr*. *ruminantium* strain M1 constituted the second major phylotype in both the rumen and cecum of lichen-fed reindeer. The relative proportion of *Mbr*. *ruminantium* was, however, not observed to be significantly different between both groups of reindeer, maybe because of the big inter-individual variations observed in the lichen-fed group (Rumen: p = 0.054; cecum: p = 0.258). Considering that *Mbr*. *smithii* and *Mbr*. *olleyae* represented only a 2.9% and 2.6% of the total rumen archaeal sequences in reindeer fed pellets or lichens, respectively, it may seem difficult to explain predicted lower methane yields with the intake of lichens just based on the fluctuation of these two methanogens.

*Mbr*. *wolinii* and *Mbr*. *boviskoreanis* (≥97% similarity) were significantly reduced in the cecum of reindeer fed lichens (Fig 2; S5 and S7 Files). These two methanogens are phylogenetically related [73]; an aspect that may explain the similar effect on their relative proportions with lichens. *Mbr*. *wolinii*-related phylotypes markedly increased with the intake of a tannin-rich oak leaves-based diet in sika deer [74], which were hypothesized to emit less methane than Sika deer fed corn stalk. Alternatively, a higher proportion of *Mbr*. sp. AbM4, closely related to *Mbr*. *wolinii*, was also described in the rumen of cattle possessing low feed efficiencies, which were predicted to possess high methane yields [67]. Considering the disparity observed in both studies and the fact that cecum methanogenesis represents only a minor fraction of the total methane output from ruminants, the decrease in the relative proportion of these two methanogens would only account for a minor part of predicted reduced methane emissions with the intake of lichens. Nonetheless, the role played by these two methanogens on the overall methane output from ruminants would worth further investigation.

PICRUSt analysis showed dissimilarities in the relative abundance of genes involved in several KEGG pathways, which concurred with significant differences of bacterial and archaeal profiles between both groups of reindeer. Higher relative abundances for pyruvate and fatty acids (propionate and butyrate) metabolism-related genes were obtained in reindeer fed lichens (Fig 5a). Given that the concentration of rumen VFAs (propionate, acetate and n-butyrate) remained unaltered between both groups of reindeer (S3 Table), it was surprising to obtain such differences. In addition, significantly lower relative gene contents for ascribed genes involved in methane metabolism were found in reindeer fed lichens (Fig 5b). Deep metagenomics and metatranscriptomics studies, comparing microbial diversity and genetic profiles between high- and low-CH_4_ emitting sheep, demonstrated similar gene contents for KEGG pathways involved in methanogenesis [75]. Instead, higher expression levels for some of these genes were reported for the animals with high methane yields. Based on these findings, the differences observed between reindeer fed pellets or lichens may not necessarily predict for lower methane yields with a lichen-based diet. It would demand the use of more specific approaches (metatranscriptomics) in order to complement the results obtained in our study and assess for differences in the expression level of these genes involved in methanogenesis, which would allow elucidating potential changes in the methane output with the ingestion of lichens. The PICRUSt analysis represent a valuable first look at the respective genetic profiles from both groups of reindeer in order to guide future analysis.

In conclusion, the present study demonstrated that both the bacterial and archaeal microbiomes housed in the rumen and cecum of Norwegian reindeer was altered in response to the intake of a mixed lichen diet. As discussed, several factors might lead to such fluctuations, though reported high PSM contents in lichens is hypothesized to be the main driving force. These results are a valuable complement to previous findings addressing the use of diets high in PSM, or extracted compounds, envisaged as a potential strategy for enteric methane mitigation. The outstanding tolerance demonstrated by Norwegian reindeer to a PSM-rich diet certainly stands for its potential as a candidate to conduct more research focused on understanding the relationship between the effects exerted by these plant anti-nutrients on the archaeal community structure, and how this is linked with predicted lower methanogenesis. Nonetheless, a direct estimation on the methane yields from reindeer fed lichens would be the best way to determine differences in the methane output between both feeding regimes, and it would be a valuable support to the findings presented in this study. More research is paramount to pinpoint the community structures displayed by the other syntrophic partners of methanogens co-habiting the rumen and cecum, such as ciliate protozoa or fungi, under similar feeding regimes. This would certainly allow for a broader view on the metagenomic profiles specific for those conditions when low methane outcomes are obtained.

## Supporting Information

S1 File Quantitative real-time PCR conditions for bacteria, archaea and protozoa in rumen and cecum samples from Norwegian reindeer. (XLSX)

S2 File Bacterial diversity and average phylotype percentages for rumen and cecum samples from pellets and lichens fed reindeer. (XLSX)

S3 File Stacked plots of bacterial diversity in rumen samples from Norwegian reindeer. (A) Classification of bacteria at phylum level. (B) Classification of bacteria up to genus level.
(PDF)

S4 File Stacked plots of bacterial diversity in cecum samples from Norwegian reindeer. (A) Classification of bacteria at phylum level. (B) Classification of bacteria up to genus level.
(PDF)

S5 File Archaeal diversity and average phylotype percentages for rumen and cecum samples from pellets and lichens fed reindeer. (XLSX)

S6 File Stacked plots of archaeal diversity in rumen samples from Norwegian reindeer. (A) Classification of bacteria at phylum level. (B) Classification of bacteria up to genus level.
(PDF)

S7 File Stacked plots of archaeal diversity in cecum samples from Norwegian reindeer. (A) Classification of bacteria at phylum level. (B) Classification of bacteria up to genus level.
(PDF)

S1 Table Bacterial alpha diversity parameters for rumen and cecum samples from Norwegian reindeer. (DOCX)

S2 Table Archaeal alpha diversity parameters for rumen and cecum samples from Norwegian reindeer. (DOCX)

S3 Table Concentration of the main short-chain fatty acids found in rumen samples from Norwegian reindeer. (DOCX)
