# Supplementary figures and images for: Rumen and Cecum Microbiomes in Reindeer (Rangifer tarandus tarandus) Are Changed in Response to a Lichen Diet and May Affect Enteric Methane Emissions
Source: PLoS One. 2016 May 9;11(5):e0155213. doi: 10.1371/journal.pone.0155213 (PMC4861291; doi:10.1371/journal.pone.0155213)

**a.**

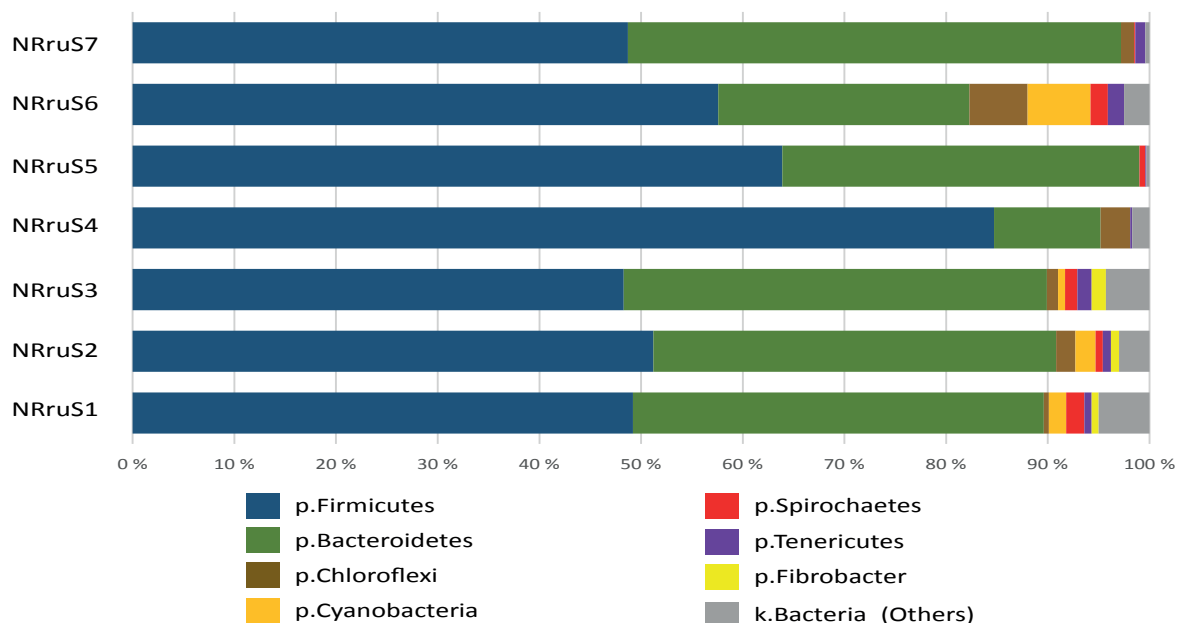

**b.**

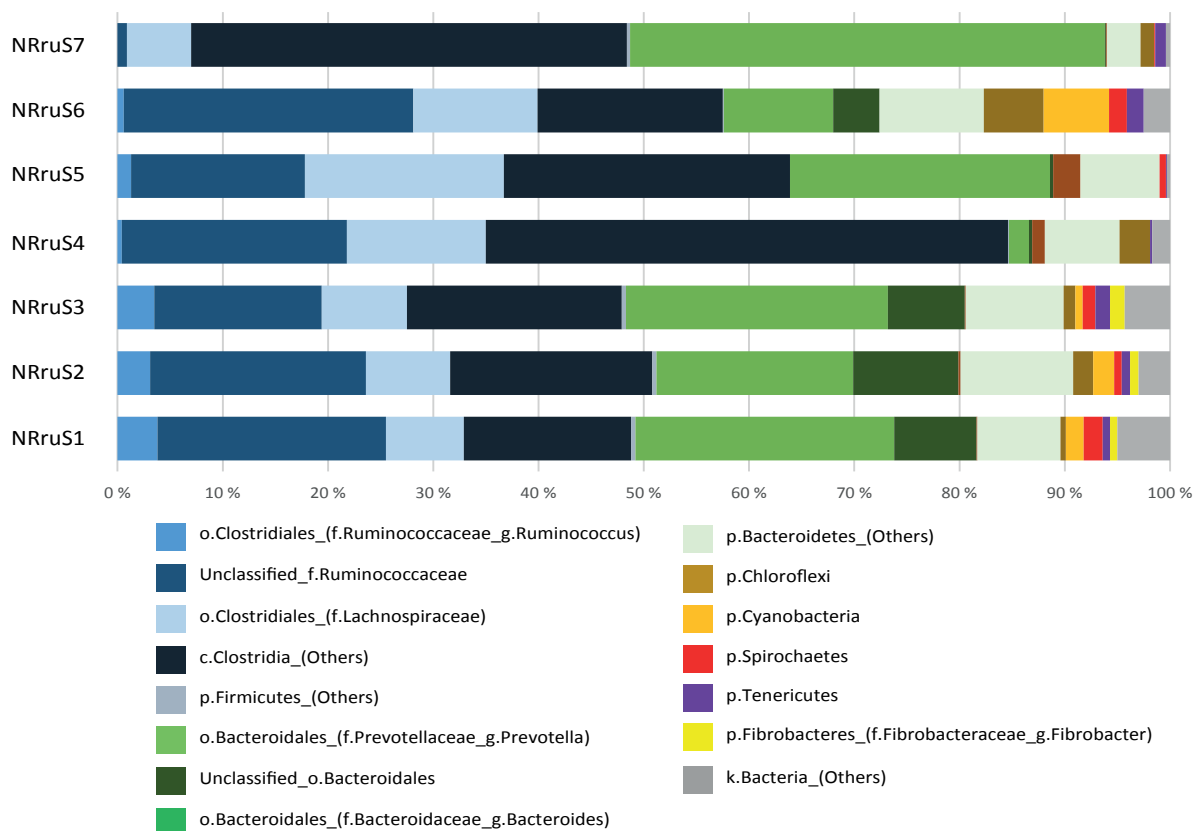

Supplement: S3 File — (A) Classification of bacteria at phylum level. (B) Classification of bacteria up to genus level. (PDF) [file pone.0155213.s003.pdf]

**a.**

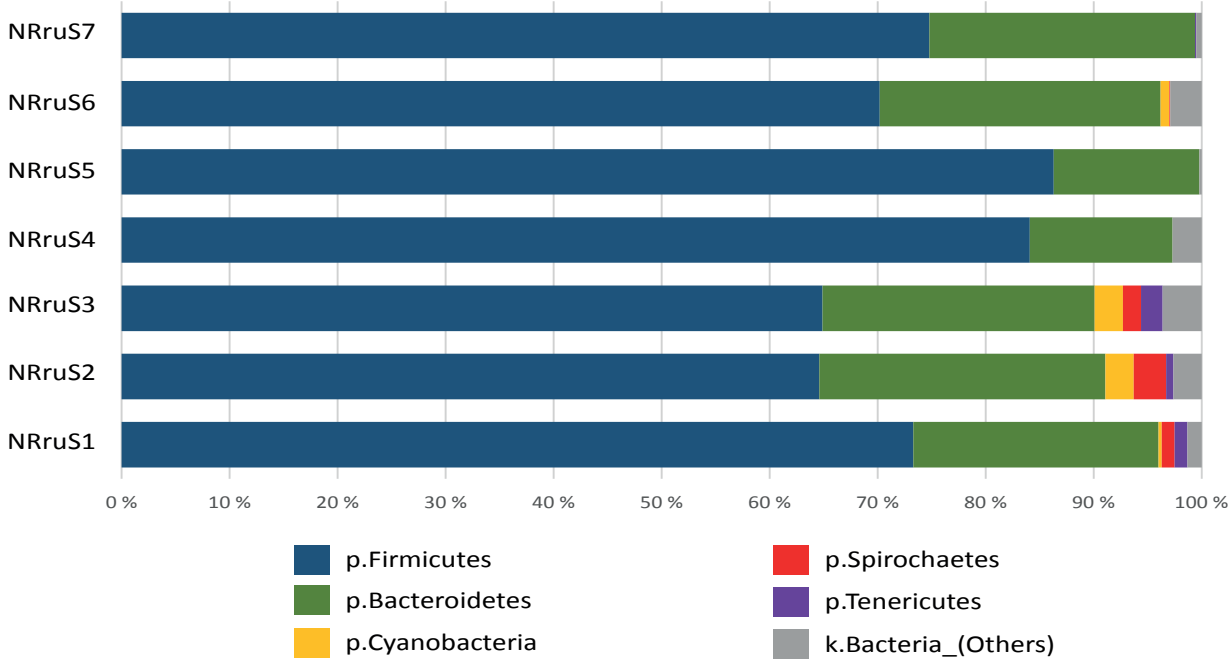

**b.**

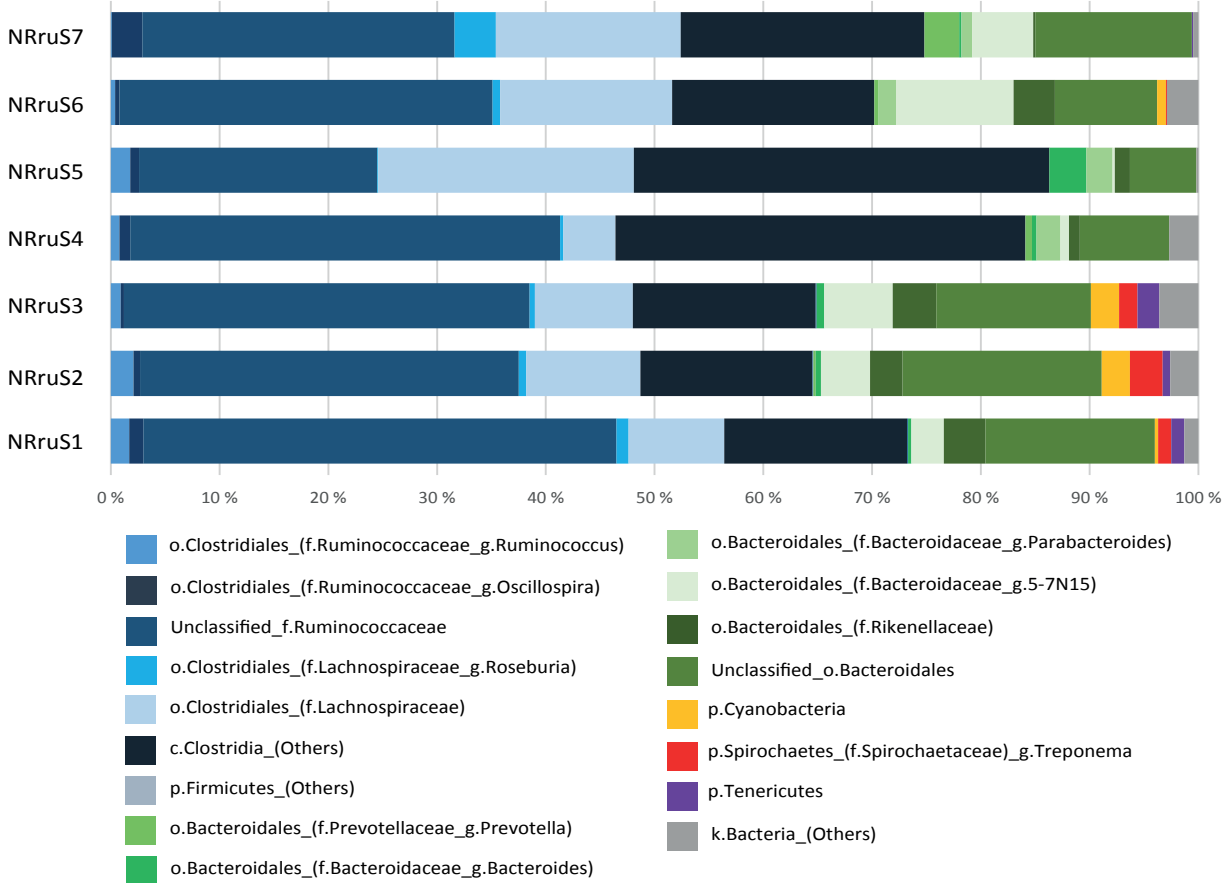

Supplement: S4 File — (A) Classification of bacteria at phylum level. (B) Classification of bacteria up to genus level. (PDF) [file pone.0155213.s004.pdf]

**a.**

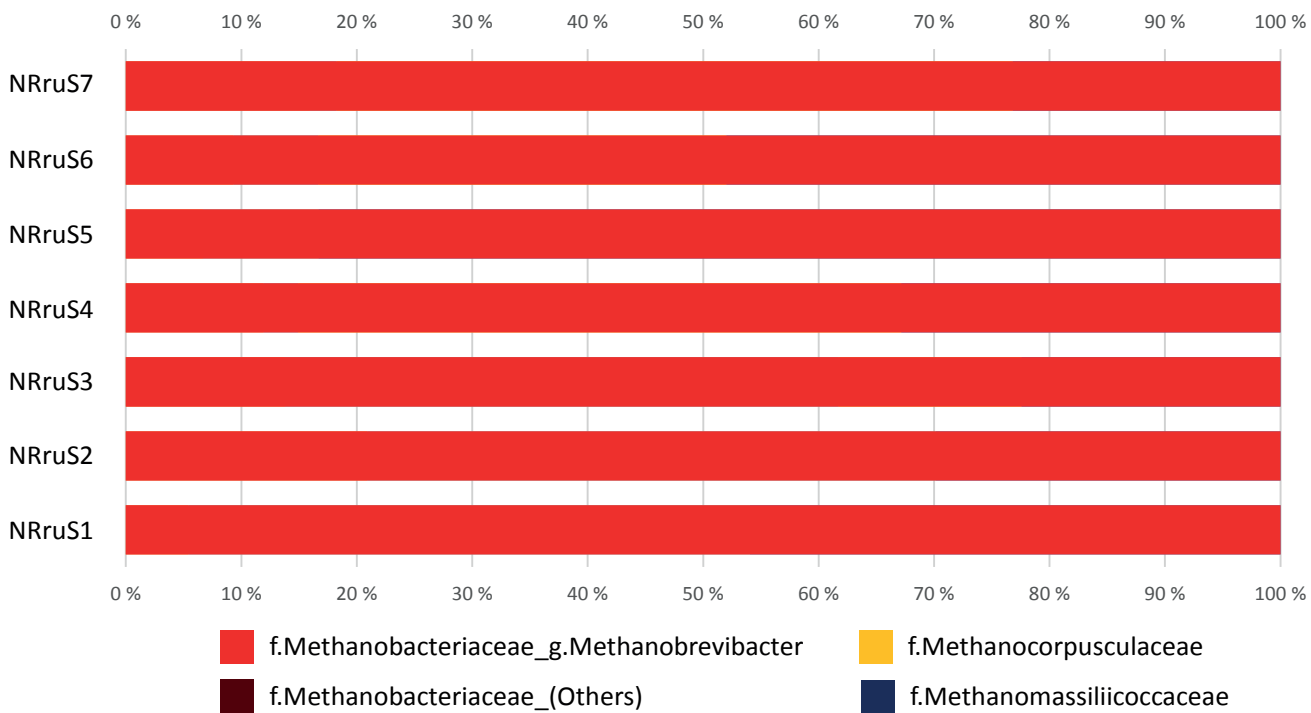

**b.**

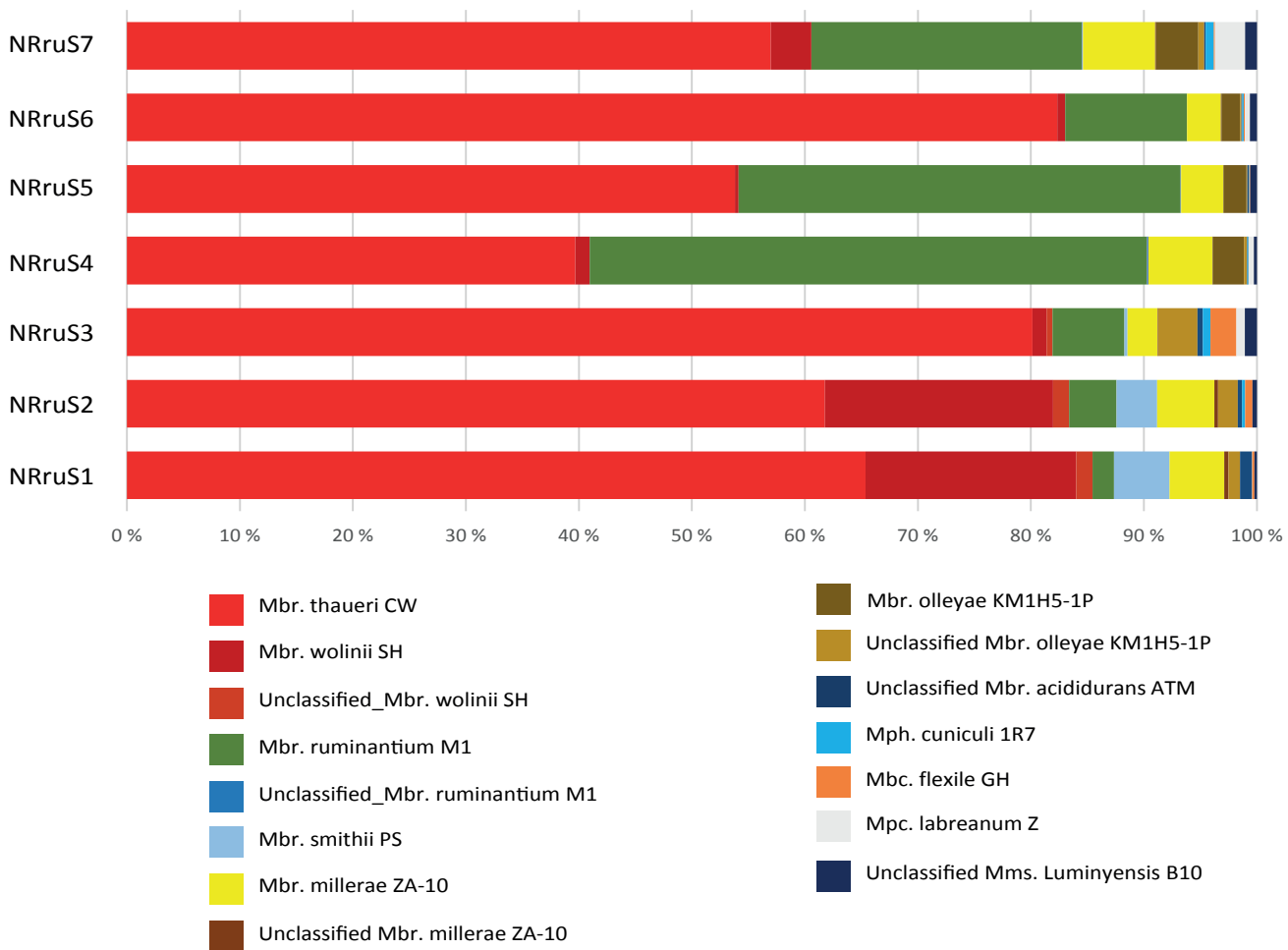

Supplement: S6 File — (A) Classification of bacteria at phylum level. (B) Classification of bacteria up to genus level. (PDF) [file pone.0155213.s006.pdf]

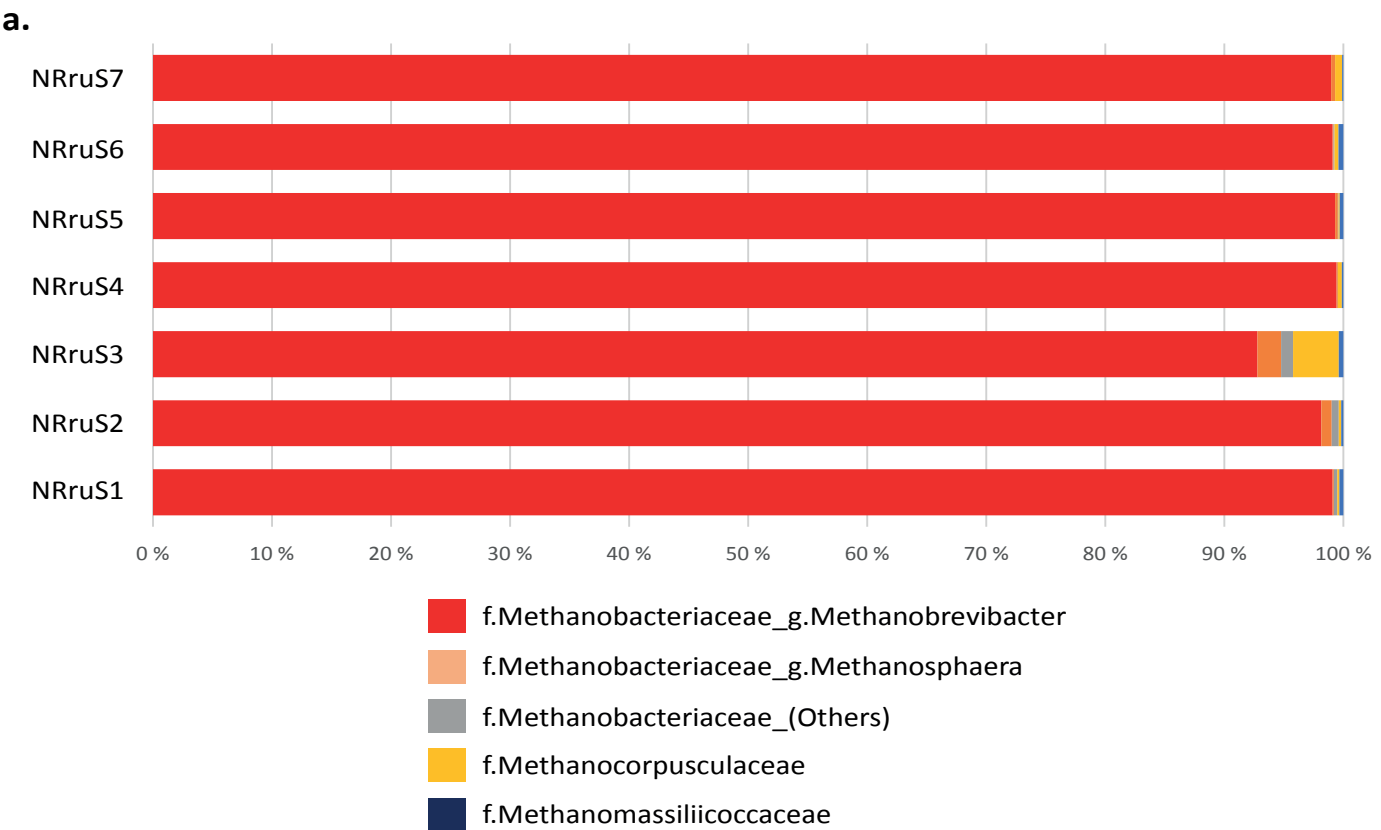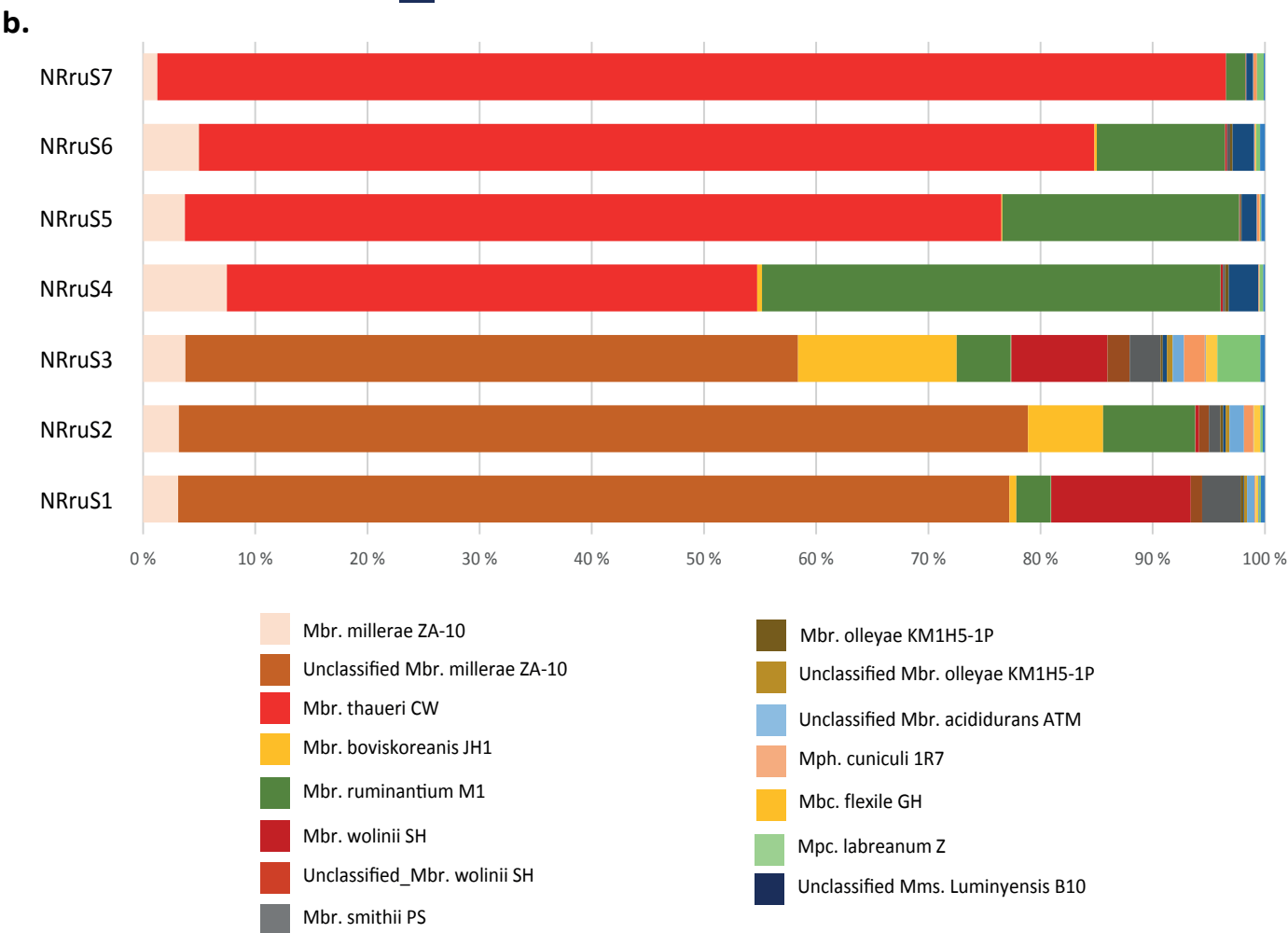

Supplement: S7 File — (A) Classification of bacteria at phylum level. (B) Classification of bacteria up to genus level. (PDF) [file pone.0155213.s007.pdf]
