# Supplementary material for: Rumen and Cecum Microbiomes in Reindeer (Rangifer tarandus tarandus) Are Changed in Response to a Lichen Diet and May Affect Enteric Methane Emissions
Source: PLoS One. 2016 May 9;11(5):e0155213. doi: 10.1371/journal.pone.0155213 (PMC4861291; doi:10.1371/journal.pone.0155213)
Supplement: S1 Table — (DOCX) [file pone.0155213.s008.docx]

| **Sample ID** | **Chao1^a,b^** | **Shannon-Wienner index ^a,b^** | **Observed species ^a,b^** | **Good’s coverage** |
| --- | --- | --- | --- | --- |
| NRruS1 | 1152 | 8.48 | 707 | 0.81 |
| NRruS2 | 1192 | 8.78 | 736 | 0.81 |
| NRruS3 | 1195 | 8.48 | 707 | 0.81 |
| NRruS4 | 272 | 5.16 | 193 | 0.96 |
| NRruS5 | 537 | 5.98 | 255 | 0.95 |
| NRruS6 | 407 | 6.36 | 274 | 0.94 |
| NRruS7 | 90 | 2.79 | 64 | 0.98 |
| NRceS1 | 697 | 8.14 | 498 | 0.91 |
| NRceS2 | 616 | 8.39 | 548 | 0.90 |
| NRceS3 | 660 | 8.31 | 528 | 0,91 |
| NRceS4 | 273 | 5.58 | 194 | 0.96 |
| NRceS5 | 347 | 6.27 | 261 | 0.95 |
| NRceS6 | 385 | 6.63 | 267 | 0.94 |
| NRceS7 | 18 | 5.79 | 201 | 0.96 |

^a^ Statistical significance between samples collected from the rumen of reindeer fed pellets (NRruS1-S3) and lichens (NRruS4-S7).

^b^ Statistical significance between samples collected from the cecum of reindeer fed pellets (NRruS1-S3) and lichens (NRruS4-S7).
