# Supplementary material for: Rumen and Cecum Microbiomes in Reindeer (Rangifer tarandus tarandus) Are Changed in Response to a Lichen Diet and May Affect Enteric Methane Emissions
Source: PLoS One. 2016 May 9;11(5):e0155213. doi: 10.1371/journal.pone.0155213 (PMC4861291; doi:10.1371/journal.pone.0155213)
Supplement: S2 Table — (DOCX) [file pone.0155213.s009.docx]

| **Sample ID** | **Chao1** | **Shannon-Wienner index** | **Observed species** | **Good’s coverage** |
| --- | --- | --- | --- | --- |
| NRruS1 | 33 | 1.68 | 31 | 0.99 |
| NRruS2 | 47 | 1.71 | 35 | 0.99 |
| NRruS3 | 37 | 2.57 | 36 | 0.99 |
| NRruS4 | 31 | 1.96 | 26 | 0.99 |
| NRruS5 | 38 | 1.33 | 23 | 0.99 |
| NRruS6 | 37 | 1.3 | 28 | 0.99 |
| NRruS7 | 48 | 2.52 | 40 | 0.99 |
| NRceS1 | 33 | 1.96 | 31 | 0.99 |
| NRceS2 | 39 | 1.88 | 34 | 0.99 |
| NRceS3 | 37 | 2.87 | 35 | 0.99 |
| NRceS4 | 30 | 2.02 | 25 | 0.99 |
| NRceS5 | 35 | 1.52 | 19 | 0.99 |
| NRceS6 | 33 | 1.39 | 27 | 0.99 |
| NRceS7 | 20 | 0.69 | 13 | 0.99 |
