# Supplementary material for: Rumen and Cecum Microbiomes in Reindeer (Rangifer tarandus tarandus) Are Changed in Response to a Lichen Diet and May Affect Enteric Methane Emissions
Source: PLoS One. 2016 May 9;11(5):e0155213. doi: 10.1371/journal.pone.0155213 (PMC4861291; doi:10.1371/journal.pone.0155213)
Supplement: S3 Table — (DOCX) [file pone.0155213.s010.docx]

| **Sample ID** | **Acetate (mg/mL)** | **Propionate (mg/mL)** | **n-Butyrate (mg/mL)** | **Acetate:propionate** |
| --- | --- | --- | --- | --- |
| NRruS1 | 48.8 | 51.9 | 2.9 | 0.9 |
| NRruS2 | 22.2 | 57.4 | 6.6 | 0.4 |
| NRruS3 | 35.3 | 35.3 | 2.5 | 1.0 |
| NRruS4 | 18.8 | 7.1 | 13.4 | 2.6 |
| NRruS5 | 31.9 | 9.6 | 0.5 | 3.3 |
| NRruS6 | 41.6 | 18.9 | 0.9 | 2.2 |
